# Residue Analysis and Dietary Risk Assessment of Pymetrozine in Potato (*Solanum tuberosum* L.) and *Chrysanthemum morifolium* (Ramat)

**DOI:** 10.3390/plants12223905

**Published:** 2023-11-20

**Authors:** Yuting Chen, Hui Ye, Nan Fang, Yuqin Luo, Xiangyun Wang, Yanjie Li, Hongmei He, Youpu Cheng, Changpeng Zhang

**Affiliations:** 1 School of Horticulture and Landscape Architecture, Tianjin Agricultural University, Tianjin 300380, China; 2 Institute of Agro-Products Safety and Nutrition, Zhejiang Academy of Agricultural Sciences, Hangzhou 310021, China; fn199198@hotmail.com (N.F.); 13665805502@163.com (Y.L.); wangxiangyun2@sina.com (X.W.); yanjieli0913@outlook.com (Y.L.); hehongmei@163.com (H.H.)

**Keywords:** risk assessment, Potato (*Solanum tuberosum* L.), *Chrysanthemum morifolium* (ramat), pymetrozine, HPLC-MS/MS

## Abstract

Pymetrozine is used on potato (*S. tuberosum*) and *Chrysanthemum morifolium* (*C. morifolium*) to obtain greater yield and quality. However, pesticide use carries the potential for residues to remain and be detected on harvested crops. Therefore, the aim of this study was to estimate pesticide residues in *S. tuberosum* and *C. morifolium* products that are commercially available for human consumption and to assess the associated dietary risks. For this study, a total of 340 samples (200 *S. tuberosum* samples and 140 *C. morifolium* samples) were collected randomly from supermarkets and farmer’s markets. Residues of pymetrozine in *S. tuberosum* and *C. morifolium* were detected by using an established and validated QuECHERS-HPLC-MS / MS method, while a dietary risk assessment of pymetrozine in *S. tuberosum* and *C. morifolium* was performed using these data. The detection rates of pymetrozine in *S. tuberosum* and *C. morifolium* samples were 92.31% and 98.17%, respectively, with residues not more than 0.036 and 0.024 mg/kg, respectively. Based on these results, the dietary risk assessment indicated that the intake of pymetrozine residues in *S. tuberosum* and *C. morifolium* does not pose a health risk. This work improved our understanding of the potential exposure risk of pymetrozine in *S. tuberosum* and *C. morifolium*.

## 1. Introduction

Potato (*S. tuberosum*) is the fourth largest food crop after maize, wheat and rice [1]. China is the country with the largest *S. tuberosum* cultivation area in the world. China is the fourth largest potato exporter in the world; its potato exports account for 6% of the world’s *S. tuberosum* exports. From 2011 to 2021, the output of *S. tuberosum* increased from 18.309 million tons to 21.055 million tons in China [2]. *C. morifolium* is an important cultivar of medicinal and edible chrysanthemum floss, which is widely planted in different regions of China [3]. It is particularly popular with the Chinese as it not only protects the heart and blood vessels but also has weight loss, anti-fatigue and digestive effects [4,5,6]. However, *S. tuberosum* and *C. morifolium* have suffered from increased pests and diseases during the planting process, such as late blight, root rot, aphids and litura [7,8,9]. The aphid is the major pest of *S. tuberosum* and *C. morifolium* in cultivation. According to the investigation from 2008 to 2017 on the national plant protection statistical data, the yield loss due to grubs and aphids was 11.65–18.60% in the two cropping areas of the central plains for China’s four major *S. tuberosum* growing regions, with a yield loss of 10.14% due to damage by aphids in the southern winter cropping area [10]. *C. morifolium* is frequently attacked by various types of pests, including aphids, gall midges, leafhoppers and other pests, with aphid infestation covering more than 80% of the area [11,12,13]. Because *S. tuberosum* and *C. morifolium* may suffer from various types of pest damage during the planting process, various pesticides are usually applied to control the above damage for better yield and quality.

In China, pymetrozine insecticide is commonly used in *S. tuberosum* and *C. morifolium*. basic structure of a pyridine azomethine, which mainly affects the feeding behaviour of insects, causing them to die by antifeeding, and has good control efficacy against insects with piercing sucking mouthparts, Insects such as aphids [14]. Therefore, it is used as an alternative to organophosphorus pesticides to control aphids, leafhoppers, nilaparvata lugens, etc. [15,16]. However, the extensive use of pesticides may have adverse effects on the environment and health. Using the chemical footprint (ChF) method assessment to examine the ecological impact of pesticide use in China, Jing et al. [17] showed that the pesticides pymetrozine, chlorpyrifos and atrazine have a greater ecological impact on China’s ecosystems than other pesticides, although they account for less than 10% of total pesticides. In addition, previous studies found that pesticide residues in agricultural products can be transferred to humans through food consumption and contribute to adverse health, including cancer, neurodevelopmental disorders, birth defects, endocrine disruptions, etc. [18,19,20]. Meanwhile, the European Food Safety Authority identified pymetrozine as a possible human carcinogen in 2017 [20]. Considering that agricultural products are the main route by which consumers are exposed to pymetrozine, foods containing residues of this substance may pose a dietary danger. Commercially available agricultural products are the main way for consumers to be exposed to pesticides, which may pose dietary risks when eating agricultural products containing pesticide residues. Therefore, investigating the dietary risks posed by pesticide residues is essential.

However, the currently available research has concentrated on residue detection methods, dissipation behavior and risk assessment in the field regarding *S. tuberosum* and *C. morifolium*. Wang et al. [21] detected pymetrozine residues in Chinese cabbage, which showed an average residue of 0.25 mg kg^−1^. Zhang et al. [22] found that pymetrozine had the highest final residue in rice tissues of 0.002–0.003 mg kg^−1^. Yu et al. [23] compared residual pymetrozine levels in dry and fresh tea and mean residual pymetrozine levels in dry and fresh tea, which were 0.85 and 10.55 mg kg^−1^, respectively. However, studies on the dietary risk assessment and residual status of pymetrozine in market *S. tuberosum* and *C. morifolium* have not been reported in China.

Therefore, the purpose of this study is (1) to establish a method for the analysis of pymetrozine residues by using HPLC-MS/MS in *S. tuberosum* and *C. morifolium*, (2) to determine residual pymetrozine levels in actual samples collected from different regions and (3) to assess dietary risk based on the residue data.

## 2. Materials and Methods

### 2.1. Chemicals and Reagents

The standard for pymetrozine (purity > 97.7%) was obtained from Ehrenstorfer GmbH (Augsburg, Germany). Acetonitrile and methanol (chromatographic grade) were purchased from Merck (Darmstadt, Germany). Absolute alcohol and acetonitrile (analytical reagent grade) were supplied by Shanghai Lin-feng Chemistry Reagent Co. Ltd. (Shanghai, China). Sodium chloride (NaCl) and ammonia water (Mass fraction: 28%) were obtained from Sinopharm Co. Ltd. (Beijing, China). Octadecyl silica (C_18_), anhydrous magnesium sulfate (MgSO_4_) and primary secondary amines (PSA) were purchased from Tianjin Bonna-Agela Phenomenex Technologies (Tianjin, China). Water was purchased from Watsons (Hangzhou, China). The pesticides were prepared as stock solutions using methanol at a concentration of 1000 mg L^−1^ and stored at −18 °C (replaced after three months).

### 2.2. Sample Collection and Preparation

In 2022, 340 samples of *S. tuberosum* and *C. morifolium* were randomly collected from agricultural wholesale markets and supermarkets in 17 different regions according to the guideline on sampling for pesticide residue analysis (Jilin, Shangdong, Beijing, Hunan, Henan, Qinghai, Guizhou, Chongqing, Hubei, Zhejiang, Anhui, Jiansu and Gan su) of China [24]. In each region, we obtained at least 20 *S. tuberosum* and *C. morifolium* samples for a total of 200 S. tuberosum samples and 140 *C. morifolium* samples. 

All samples were collected and placed in black plastic bags to prevent contamination and spoilage, labeled and brought to the lab. S. tuberosum samples were chopped into small pieces and fully blended using the homogenizing machine. *C. morifolium* samples were ground using dry ice and an electric grinder. The homogenizing machine was thoroughly cleaned with water and absolute alcohol to avoid cross-contamination in the next sample. The samples were frozen at −20 °C until extraction for analysis. 

### 2.3. Sample Pretreatment

For extraction, 5 g of homogenized *S. tuberosum* and powdery *C. morifolium* samples was added in a 50 mL centrifuge tube, after which 10 mL of 0.1% ammonia-acetonitrile solution was added and samples were extracted by shaking for 15 min. The shaken samples were mixed with 1 g NaCl and 4 g MgSO_4_ and then centrifuged for 5 min at 4000 rpm. 

After centrifugation, 1.6 ml of the supernatant was purified in a QuECHERS tube (150 mg MgSO_4_, 50 mg PSA and 50 mg C_18_ were added to the supernatant of *S. tuberosum*, and 150 mg MgSO_4_ and 50 mg PSA were added to the supernatant of *C. morifolium*). Then, we vortexed the sample for 1 min, followed by centrifugation at 8000 rpm for 5 min. We used a 0.22 μm filter to filter the extract before HPLC-MS/MS analysis.

### 2.4. HPLC-MS/MS Analysis

The pesticide was analyzed on a Waters ultra-high performance liquid chromatography triple quadrupole mass spectrometer (HPLC-MS/MS). Pymetrozine was separated using a Waters Acquity HPLC HSS T3 analytical column (2.1 × 100 mm, 1.8 μm) at a flow rate of 0.2 mL min^−1^ and with a sample injection volume of 2 µL at 40 °C. The mobile phase was formed by using solvent A (acetonitrile) and solvent B (pure water). The elution program of the chromatographic gradient was carried out as follows: 0~2.5 min, 10% A; 2.5~3.5 min, 10~30% A; and 3.5~6.0 min, 30~10% A. The total separation time was 6.0 minutes.

Electrospray ionization (ESI+) was used for the mass spectrometric analysis of pymetrozin. The capillary voltage was set at 1.5 kV and the extractor voltage at 4 V. Multiple reaction monitoring (MRM) mode was performed to detect pymetrozine, and the quantification transition and qualitative transition values were *m*/*z* 218.03→104.85 (collision energy, 18 V) and *m*/*z* 218.03→78.39 (collision energy, 36 V), respectively. As for other parameters, the ion source temperature was set to 115 °C, the desolvation temperature was set to 450 °C, and the desolvation gas flow rate was set to 600 L h^−1^.

### 2.5. Method Validation

Linearity, the limit of detection (LOD), the limit of quantification (LOQ), accuracy, precision and the method matrix effect were evaluated in different matrices to validate the proposed QuEChERS-HPLC-MS/MS method. Analytical curves were established to determine the linearity of the method at different levels of concentration of pymetrozine: 0.001, 0.01, 0.02, 0.05, 0.1 and 0.2 mg·L^−1^, for which correlation coefficients (R^2^) were obtained according to the linear regression equation. Recovery experiments were performed on five replicate samples at three different levels to verify the accuracy of the method, expressed as the RSD (relative standard deviation) between parallel samples (*S. tuberosum*: 0.01, 0.02 and 0.05 mg kg^−1^; *C. morifolium*: 0.01, 0.05 and 0.1 mg kg^−1^). The LOQ shows the lowest detectable concentration of pymetrozine in the sample. For the improvement of the analysis for the accuracy of pymetrozine, we performed an evaluation of the matrix effect. The matrix effect calculation was as follows (1) [25]:

(1) $$\mathrm{ME}\left(\%\right)=\left(\frac{S_{m}}{S_{S}}\right)\times 100$$

where S_m_ and S_S_ are the slopes of the calibration curves for the matrix and the solvent, respectively. Matrix effects (MEs) were defined as the total influence of components in the sample other than pymetrozine on the measurement, which may inhibit or enhance the analyte signal. MEs ranging from −20% to 20% were considered low, those from −50% to 50% were considered moderate, and those <−50% or >50% were considered strong [26].

### 2.6. Risk Assessment

A dietary risk assessment of pesticides derives dietary exposure estimates from calculated food consumption data and chemical concentrations in food for the target population to achieve risk characterization [27]. The Chinese Dietary Risk Assessment Model was utilized to estimate the national estimated daily intake (NEDI) and the national estimated short-term intake (NESTI) of pymetrozine in *S. tuberosum* and *C. morifolium*. The chronic risk quotient (HQ_c_) and acute risk quotient (HQ_a_) were used to assess long-term and short-term dietary risk, respectively.

A chronic intake risk (HQ_c_) assessment for pymetrozine was conducted using Equations (2) and (3) [28,29]:

(2) $$\mathrm{NEDI}=\sum \left(\mathrm{STMRs}\times \mathrm{Fi}\right)\;$$

(3) $${\mathrm{HQ}}_{C}=\frac{\mathrm{NEDI}}{\mathrm{bw}\times \mathrm{ADI}}\times 100\%$$

where NEDI is the national estimate daily intake for the country (mg). STMRs are the median of pesticide residues (mg kg^−1^). Fi is the average daily consumption of agricultural products (kg day^−1^). The ADI of pymetrozine represents its acceptable daily intake (mg kg^−1^ bw). 

The following formula is used to calculate NESTI and HQ_a_ [30,31]:

(4) IESTI=(HR×LP)/bw

(5) $${\mathrm{HQ}}_{a}=\frac{\mathrm{IESTI}}{\mathrm{ARfD}}\times 100\%$$

where IESTI is the estimated short-term intake of pesticide residues (mg). HR is the highest residue of *S. tuberosum* and *C. morifolium* (mg kg^−1^). LP represents the largest portion (97.5th percentile) of food consumed by individuals in a day (kg day^−1^). bw is the average weight of a Chinese adult, which is 63 kg. ARfD is the acute reference dose (mg k) of pymetrozine. 

When HQ_c_ or HQ_a_ is less than 100%, the risk is considered acceptable and to not pose a long-term health or acute intake risk, whereas when HQ_c_ or HQ_a_ is greater than or equal to 100%, it is considered to pose an unacceptable long-term or acute intake risk to consumer health.

## 3. Results and Discussion

### 3.1. Analytical Method Validation

In this experiment, an external method was utilized for the quantitative analysis of pymetrozine. Table 1 shows the calibration plots for pymetrozine (0.01~0.2 mg kg^−1^), which showed good linear relationships in all matrices (R^2^ > 0.99). The LOD for pymetrozine was 0.001 mg kg^−1^, while the LOQ was 0.01 mg kg^−1^ in the matrices (Table 1). The MEs for pymetrozine ranged from 11.06% to ~12.90% (Table 1), indicating matrix enhancement. The response of the instrument was significantly enhanced by all matrices spiked with pymetrozine in the results shown. Therefore, the matrix standard solution was used to calibrate this study. 

To evaluate the accuracy and precision of the established method for the determination of pymetrozine residues in *S. tuberosum* and *C. morifolium*, the method was validated by adding 0.01, 0.02, 0.05 mg kg^−1^ and 0.01, 0.05, 0.1 mg kg^−1^ (n = 5) of pymetrozine in blank *S. tuberosum* and *C. morifolium*, respectively. The Table 2 shown that average recoveries of pymetrozine in *S. tuberosum* ranged from 89.19 to 103.57%, with an RSD of 3.02~4.27%. The average recoveries of pymetrozine in *C. morifolium* ranged from 77.26 to 106.85%, with an RSD of 2.82~4.91%. The average recovery rates for different matrices varied from 70 to 110%, with a coefficient of variation less than 5%. Overall, the recovery and RSD of the method were in line with pesticide residue analysis standards. The method validation for pymetrozine determination in *S. tuberosum* and *C. morifolium* showed that pymetrozine could be determined.

### 3.2. Pesticide Residues in Market Samples

The validated method was used to detect pymetrozine residues in 340 commercially available samples, including 200 *S. tuberosum* and 140 *C. morifolium* samples. As shown in Table 3, pymetrozine detection rates in *S. tuberosum* and *C. morifolium* samples were over 90%. The results showed that pymetrozine residues were relatively common in *S. tuberosum* and *C. morifolium* samples, suggesting that growers, consumers and government regulators should pay more attention to these issues. The detection range for *S. tuberosum* was <LOQ (0.01 mg kg^−1^) to 0.036 mg kg^−1^, and that of *C. morifolium* was <LOQ (0.01 mg kg^−1^) to 0.024 mg kg^−1^. According to the registration information, the recommended dose of pymetrozine in *S. tuberosum* (50% effective content, 20–30 g per acre) is higher than that in *C. morifolium* (25% effective content, 25–30 g per acre); moreover, pymetrozine in *S. tuberosum* is used twice a season, and *C. morifolium* is used once a season [32]. However, market sample test results showed no significant differences in mean residue levels of *S. tuberosum* (0.028 mg kg^−1^) and *C. morifolium* (0.019 mg kg^−1^), which may be due to different pymetrozine absorption and metabolism rates in different crops [33]. In addition, it may include geographical location, planting conditions and seasonal climatic factors in the collected market samples. The number of pymetrozine samples detected (98.17% *S. tuberosum*; 92.31% *C. morifolium*) was significantly higher than that not detected (1.83% *S. tuberosum*; 7.69% *C. morifolium*). Furthermore, *S. tuberosum* are tuber crops. Pesticides are partially dropped after spraying. Plants absorb pesticides from the soil and transport them, which partially enriches the stems to produce residues that may have caused the high detection rate of *S. tuberosum*. Another reason for the higher detection rate may be the widespread application of pymetrozine to protect crops.

### 3.3. Dietary Intake Risk Assessment Based on Market Surveillance Data

Pymetrozine was detected in market samples, and the risk of eating *S. tuberosum* and *C. morifolium* with pymetrozine residues to human health should be considered. The human health effects of pymetrozine were quantified as the risks of acute and chronic dietary intake. Pymetrozine assessment was defined in the Joint FAO-WHO Meeting of Pesticide Residues [34]. Therefore, only the acute and chronic dietary risks of pymetrozine were assessed. Referring to previous studies, an accurate assessment of China’s dietary patterns requires consideration of registered pymetrozine crops and corresponding MRLs from various countries [35]. In China, pymetrozine is widely used and registered in rice, wheat and other crops. Registered crops are classified by diet (Table 4). A risk probability was obtained by calculating pymetrozine NEDI and comparing it with ADI. Dietary risk assessment results are presented in Table 4.

A chronic intake risk assessment for pymetrozine residues in *S. tuberosum* and *C. morifolium* was conducted next for Chinese people (Table 4). The total NEDI was calculated using the supervised test residual median (STMR_S_) and MRLs. The STMRs of *S. tuberosum* and *C. morifolium* were 0.02 and 0.017 mg kg^−1^ from the supervised test, respectively. The ADI for pymetrozine was 0.03 mg kg^−1^ day. [34]. Consequently, the results showed that the HQ_c_ value of *S. tuberosum* and *C. morifolium* was 86.71% (less than 100%) in Chinese people, indicating that the chronic dietary risk of pymetrozine in *S. tuberosum* and *C. morifolium* was acceptable.

The acute intake risk associated with pymetrozine residues in *S. tuberosum* and *C. morifolium* was assessed next for Chinese people (Table 5). The IESTI value for pymetrozine was calculated using high-residue (HR) market samples and large portion consumption (LP) data. The HR of *S. tuberosum* and *C. morifolium* was 0.036 mg kg^−1^ and 0.024 mg kg^−1^, respectively. The LPs of *S. tuberosum* and *C. morifolium* were 0.1 kg d^−1^ and 0.043 kg d^−1^, which were used as the consumption of *S. tuberosum* and *C. morifolium* for estimates of short-term pesticide residue intake risk assessment, respectively [36,37]. JMPR [34] prescribed the pymetrozine ARfD of 0.1 mg kg^−1^ bw. Consequently, the HQ_a_ values for pymetrozine were 0.057% and 0.164% for *S. tuberosum* and *C. morifolium*, respectively. The dietary risk indices of pymetrozine detected in *S. tuberosum* and *C. morifolium* were less than 100%, indicating a safe level.

## 4. Conclusions

In this study, the establishment of a method involving the QuEChERS-HPLC-MS/MS procedure and an analysis of samples with pretreatment achieved satisfactory results in terms of linearity, analytical limits, accuracy and precision. Furthermore, the method was utilized to detect pymetrozine residues in *S. tuberosum* and *C. morifolium* samples from the market. Among the analyzed samples, the overall detection rate was over 90% (92.31% for *S. tuberosum* and 98.17% for *C. morifolium*). The results showed that pymetrozine residues were relatively common in *S. tuberosum* and *C. morifolium* samples, suggesting that growers, consumers and government regulators should pay more attention to these issues. Moreover, we conducted a dietary risk analysis to determine the consumer safety of pymetrozine residues in *S. tuberosum* and *C. morifolium*. The results showed that the dietary risk of pymetrozine in both matrices was less than 100% and that the overall risk was acceptable. Together, these results provide an important data reference into the current prevalence of pymetrozine residues in *S. tuberosum* and *C. morifolium* in the Chinese market while also providing a useful tool for the analysis of pymetrozine residues in other media.

## Figures and Tables

**Table 1:** Calibration curve, correlation coefficient (R^2^), matrix effects (MEs), limits of detection (LODs) and limits of quantification (LOQs) for pymetrozine in *S. tuberosum* and *C. morifolium*.

Matrix	Linear Range (mg L^−1^)	Calibration Curve	R^2^	MEs	LODs (mg kg^−1^)	LOQs (mg kg^−1^)
Solvent	0.001–0.2	y = 63,107,860.25x + 367,623	0.9999	/	0.001	0.01
*S. tuberosum*	0.001–0.2	y = 8,140,913.97x + 786,650	0.9997	12.90	0.001	0.01
*C. morifolium*	0.001–0.2	y = 6,979,729.34x + 140,705	0.9967	11.06	0.001	0.01

**Table 2:** Recoveries, average recoveries (ARs) and relative standard deviations (RSDs) of pymetrozine in *S. tuberosum* and *C. morifolium,* each spiked at different concentration levels.

Matrix	Spiking Levels (mg kg^−1^)	Recovery (%)					ARs (%)	RSDs (%)
		1	2	3	4	5		
*S. tuberosum*	0.01	92.1	83.51	87.16	84.11	90.55	89.19	4.27
	0.02	102.64	106.21	107.06	103.11	98.85	103.57	3.13
	0.05	98.44	96.21	109.46	96.65	105.91	101.34	3.02
*C. morifolium*	0.01	80.25	82.5	76.01	73.21	75.32	77.26	4.33
	0.05	98.85	113.41	104.38	110.55	107.1	106.85	4.91
	0.1	89.58	91.31	93.48	94.49	96.83	93.14	2.82

**Table 3:** Pymetrozine residue information of *S. tuberosum* and *C. morifolium* in different regions.

Matrix	Area	Detection /Total Samples	Total Detection rates (%)	High Residue (mg kg ^−1^)	Median Residues (mg kg^−1^)	Mean Residues ± SD (mg kg ^−1^)
*S. tuberosum* *	Changchun	19/20	98.17	0.036	0.02	0.028 ± 0.0029
	Jinan	19/20				
	Tongzhou	20/20				
	Changsha	20/20				
	Xining	20/20				
	Guiyan	18/20/				
	Xiaoxia	20/20				
	Yinchua	20/20				
	Lanzhou	20/20				
	Nanning	20/20				
*C. morifolium*	Tongxiang	20/20	92.31	0.024	0.017	0.019 ± 0.0011
	Huangshan	18/20				
	Huanggang	19/20				
	Yongzhou	19/20				
	Linyi	19/20				
	Yancheng	19/20				
	Yunyang	20/20				

* Detection samples mean the pymetrozine concentration in the samples exceeds the quatitation limit of 0.01 mg kg^−1^.

**Table 4:** The long-term dietary intake risk assessment of pymetrozine based on Chinese dietary patterns.

Food Classification	FI (kg d^−1^)	Commodity	Reference Limit (mg kg^−1^)	Sources	NEDI (mg)	ADI (mg)	HQ_C_ (%)
Rice and its products	0.2399	Brown rice	0.2	China	0.004798	0.03 × 63	
Flour and its products	0.1385	Wheat	0.02	China	0.00277		
Other cereals	0.0233	Maize	0.05	AU	0.001165		
Potatoes	0.0495	*S. tuberosum*	0.02	STMRs	0.000495		
Dark vegetables	0.0915	Spinach	15	China	1.373		
Light vegetables	0.1837	Cucumber	1	China	0.1873		
Fruits	0.0457	Peach	0.05	AU	0.02285		
Vegetable oil	0.0327	Cotton seed	0.1	China	0.00327		
Salt	0.012	*C. morifolium*	0.017	STMRs	0.00020		
Total	0.8168				1.6389	1.89	86.71

Fi is the dietary reference intake for a specific food type, which is used to plan and evaluate nutrient intakes for healthy Chinese people. The unregistered food classification is dried beans and their products, pickles, nuts, livestock and poultry, milk and its products, egg and its products, fish and shrimp, animal oil, sugars, starch and soy sauce.

**Table 5:** The acute dietary intake risk assessment of pymetrozine based on Chinese dietary patterns.

Matrix	LP (kg/day)	IESTI (mg)	HQ_a_ (%)
*S. tuberosum*	0.1	0.000057	0.057
*C. morifolium*	0.043	0.001032	0.164

## Data Availability

Data are contained within the article.
